# Is neuromuscular electrical stimulation effective for improving pain, function and activities of daily living of knee osteoarthritis patients? A randomized clinical trial

**DOI:** 10.1590/S1516-31802013000100017

**Published:** 2013-04-01

**Authors:** Aline Mizusaki Imoto, Maria Stella Peccin, Lucas Emmanuel Pedro de Paiva Teixeira, Kelson Nonato Gomes da Silva, Marcelo Abrahão, Virgínia Fernandes Moça Trevisani

**Affiliations:** I PhD. Postgraduate Student, Universidade Federal de São Paulo (Unifesp), São Paulo, Brazil.; II PhD. Professor of the Postgraduate Program on Internal Medicine and Therapeutics, Universidade Federal de São Paulo (Unifesp), São Paulo, and Head of Department of Movement Sciences, Universidade Federal de São Paulo, Campus Baixada Santista, São Paulo, Brazil.; III PhD. Postgraduate Student, Universidade Federal de São Paulo (Unifesp), São Paulo, Brazil.; IV MSc. Postgraduate Student, Universidade Federal de São Paulo (Unifesp), São Paulo, Brazil.; V PhD. Professor of the Postgraduate Program on Internal Medicine and Therapeutics, Universidade Federal de São Paulo (Unifesp), São Paulo, and Titular Professor of the Discipline of Rheumatology, Universidade de Santo Amaro (Unisa), São Paulo, Brazil.

**Keywords:** Osteoarthritis, Knee, Electric stimulation therapy, Rehabilitation, Exercise therapy, Clinical trial, Osteoartrite, Joelho, Terapia por estimulação elétrica, Reabilitação, Terapia por exercício, Ensaio clínico

## Abstract

**CONTEXT AND OBJECTIVE:**

Neuromuscular electrical stimulation (NMES) has been used in rehabilitation protocols for patients suffering from muscle weakness resulting from knee osteoarthritis. The purpose of the present study was to assess the effectiveness of an eight-week treatment program of NMES combined with exercises, for improving pain and function among patients with knee osteoarthritis.

**DESIGN AND SETTING:**

Randomized clinical trial at Interlagos Specialty Ambulatory Clinic, Sao Paulo, Brazil.

**METHODS:**

One hundred were randomized into two groups: NMES group and control group. The following evaluation measurements were used: numerical pain scale from 0 to 10, timed up and go (TUG) test, Lequesne index and activities of daily living (ADL) scale.

**RESULTS:**

Eighty-two patients completed the study. From intention-to-treat (ITT) analysis comparing the groups, the NMES group showed a statistically significant improvement in relation to the control group, regarding pain intensity (difference between means: 1.67 [0.31 to 3.02]; P = 0.01), Lequesne index (difference between means: 1.98 [0.15 to 3.79]; P = 0.03) and ADL scale (difference between means: -11.23 [-19.88 to -2.57]; P = 0.01).

**CONCLUSION:**

NMES, within a rehabilitation protocol for patients with knee osteoarthritis, is effective for improving pain, function and activities of daily living, in comparison with a group that received an orientation program. CLINICAL TRIAL REGISTRATION: ACTRN012607000357459.

## RESUMO

**CONTEXTO E OBJETIVO:**

A estimulação elétrica neuromuscular (EENM) tem sido incluída em protocolos de reabilitação de pacientes com fraqueza muscular decorrente da osteoartrite do joelho. O objetivo do presente estudo foi determinar a efetividade de um tratamento de oito semanas de EENM combinado com exercícios na melhora da dor e função em pacientes com osteoartrite do joelho.

**TIPO DE ESTUDO E LOCAL:**

Ensaio clínico randomizado realizado no Ambulatório de Especialidades de Interlagos, São Paulo, Brasil.

**MÉTODOS:**

Cem pacientes foram randomizados em dois grupos: Grupo EENM (GEENM) e grupo controle (GC). As medidas de avaliação utilizadas foram: escala numérica de dor (END) 0 a 10, teste *timed up and go* (TUG), índice de Lequesne e escala de atividades de vida diária (EAVD).

**RESULTADOS:**

Oitenta e dois pacientes completaram a pesquisa. Por meio da análise por intenção de tratar, na comparação entre grupos, o GEENM apresentou melhora estatisticamente significante em comparação ao GC na redução da dor (diferença entre as médias: 1,67 [0,31; 3,02], P = 0,01), no índice de Lequesne (diferença entre as médias: 1,98 [0,15; 3,79] e na EAVD (diferença entre as médias: -11,23 [-19,88; -2,57], P = 0,01).

**CONCLUSÃO:**

A EENM, quando inserida em um protocolo de reabilitação para pacientes com osteoartrite do joelho, é efetiva na redução da dor, melhora da função e das atividades de vida diária quando comparada a um grupo submetido a programa de orientação. REGISTRO DE ENSAIO CLÍNICO: ACTRN012607000357459.

## INTRODUCTION

Osteoarthritis is a major musculoskeletal condition characterized by loss of joint cartilage.^1^ Symptomatic knee osteoarthritis affects 12% of individuals aged 60 years and over and, for many, remains a major source of pain and functional limitation.^1^ In addition to pain, patients with knee osteoarthritis also report a sensation of instability, which can lead to falls and functional disability, thereby increasing the risk of morbidity and mortality.^2^ Some authors have reported that quadriceps weakness is associated with poorer self-reported ratings of function and disability. Since the quadriceps muscle assists in shock absorption in the knee joint, weakness in this muscle group results in greater physical stress and therefore increases the pressure on the knee.^3^

Functional assessments on patients with knee osteoarthritis can be performed using a functionality questionnaire or performance tests in which the patient is observed and evaluated.^4^ The main functional questionnaires used to assess patients with knee osteoarthritis include the Western Ontario and McMaster Universities (WOMAC) index, Lequesne index and activities of daily living (ADL) scale.^5^^,^^6^^,^^7^ Among the functional performance tests used for patients with knee osteoarthritis, the most popular and reproducible of these are the timed up and go (TUG) test, six-minute walking test, get up and go test and stair climbing test.^8^^,^^9^ These forms of functional assessment (self-reported and performance tests) are considered to be reproducible at low cost, and are very commonly used tests for patients with knee osteoarthritis.^9^

Neuromuscular electrical stimulation (NMES) is defined as application of an electrical current to the neuromuscular junction and surrounding muscle fibers in order to produce a visible muscle contraction due to activation of intramuscular nerve branches.^10^ NMES can be used for: (1) preservation of muscle mass and function during prolonged periods of disuse or immobilization; (2) recovery of muscle mass and function following prolonged periods of disuse or immobilization; (3) improvement of muscle function in different healthy populations: elderly subjects, recreational athletes and competitive athletes; and (4) preoperative strengthening.^10^

It has been suggested that NMES should be used in combination with traditional strengthening programs.^11^ The effect of NMES is believed to occur through increasing the capacity of the muscle to generate force.^11^

The methods and results of the several previous studies on knee osteoarthritis differ from each other specifically in relation to NMES stimulation parameters. These have varied in frequency (25 to 50 Hz), lengths of time on and off (5 to 10 seconds), duration of NMES application (4 to 12 weeks) and setting of NMES application (at home or in a secondary care facility).^12^^,^^13^^,^^14^^,^^15^^,^^16^ This has resulted in a lack of consensus regarding the inclusion of NMES in rehabilitation protocols.

Important method flaws have been observed when assessing the methodological quality of previous studies that investigated NMES in patients with knee osteoarthritis. Thus, due to the divergences in methods, results and methodological quality among the previous studies, the aim in the present study was to conduct a clinical trial with improved methodological quality.

Since functional limitations are the main factors giving rise to impairments among patients with knee osteoarthritis, it is therefore essential to research interventions that have the aim of improving mobility and therefore functionality in this particular population of patients.^4^ Our hypothesis was that patients who received NMES would achieve improvements in pain and function, compared with patients only receiving guidance.

## OBJECTIVE

The purpose of the present randomized clinical trial was to assess the effect of NMES, as shown by the Numerical Rating Scale (NRS), TUG test, Lequesne index and ADL scale, regarding improvements among patients with knee osteoarthritis.

## METHODS

The present study was conducted in a secondary care facility, the Theumatology Department of Interlagos Specialty Ambulatory Clinic, São Paulo, Brasil, where the patients endolled were registered. This study was registered in the Australian Clinical Trials Registry (number: ACTRN012607000357459). The Ethics Committee of the Federal University of São Paulo (Universidade Federal de São Paulo, Unifesp), Brazil, approved the present study (study registration number: CEP 0141/07). To report on this randomized clinical trial, the authors followed the recommendations of the CONSORT (Consolidated Standards of Reporting Trials) Statement.^17^

A statistician generated a random allocation sequence, and simple randomization was performed by using a random-generator on a computer. To avoid selection bias, an impartial person numbered and sealed the opaque envelopes.

### Sample size

A priori power analysis calculation established that a sample size of 40 subjects per group would provide 80% power to detect a meaningful clinical difference in a TUG test of one second (SD of three seconds), with pairwise comparison among the three groups at an alpha level of 0.05 (two-tailed test), using analysis of covariance (ANCOVA) in which the covariate of the baseline measurements of the TUG test was obtained through previous studies.^18^

### Participants

One hundred patients were selected from the Rheumatology Department registers. The inclusion criteria were that the patients should present: ages ranging from 50 to 75 years; a diagnosis of knee osteoarthritis according to the criteria established by the American College of Rheumatology (ACR) using history, physical examination and radiographic findings, knee X-rays in the last 12 months; and osteoarthritis grade 2 or more based on the radiographic classification developed by Kellgren and Lawrence. The exclusion criteria were: use of a pacemaker, unstable cardiac status, attendance in a physical activity program more than twice a week (to avoid influence on the protocol to be tested), inability to ride a stationary bicycle, inability to walk and previous knee arthroplasty. After the screening procedures, the patients were assigned to one of two different groups. The groups were as follows: 1) neuromuscular electrical stimulation group (NMES group; n = 50); and 2) control group (n = 50).

### Patient medication

The patients’ medications were standardized and remained unchanged during the treatment. Paracetamol was the drug prescribed for pain and diacerein and chloroquine were used to control osteoarthritis.

### Interventions

All patients in both groups (NMES group and control group) received an educational guide (Annex 1) and were instructed to use ice packs if they had any swelling of the knee and hot packs if they had any pain without inflammation.

### NMES group

The NMES group received an educational guide and underwent quadriceps strengthening exercises and simultaneous NMES treatment. Each patient was seated on a chair, with 90 degrees of hip and knee flexion. The patient was instructed to perform a contraction of the quadriceps whenever NMES was received. An ankle weight was used to test the muscle and provide resistance during knee extension. The strengthening exercise was based on 50-60% of a test using 10 maximum repetitions, instead of a single maximum repetition, in order to avoid the possibility of injury caused by excessive strain.^19^ According to the tolerance shown by the patient, the weight load could be increased. The total duration of the sessions was approximately 40 minutes, which included 10 minutes on the exercise bike and hamstring stretching (three times of 30 seconds on each leg) before each NMES treatment.

For NMES application, two self-adhesive electrodes (Valotrude self-adhesive electrode; 7.5 x 13 cm) were positioned on the rectus femoral and vastus medialis muscles. The parameters used were: frequency of 50 Hz; pulse duration of 250 microseconds; time on: 10 seconds; and time off: 30 seconds, for 20 minutes. The intensity of the NMES used was the maximum tolerated by each patient, although this intensity was not recorded. The waveform used was pulsed rectangular biphasic and symmetrical. The equipment used was the ACTIVA 600 (Globus do Brasil Tecnologia Avançada Ltda). Use of rectangular, biphasic pulsed current with a pulse duration ranging from 100 to 400 microseconds and stimulation frequency of 50-100 Hz,^20^ at the maximum intensity tolerated by the patient and with a treatment period ranging from 3 to 12 weeks, is recommended.^21^

### Control group

The educational guide **(**Annex 1**)** was explained verbally and provided as a written explanation for the control group at the beginning of the eight-week period. During this period, the patients received two phone calls to encourage them to follow the educational guidelines. The purpose of the guide was to describe knee osteoarthritis and advise the patients on how to adjust to daily activities, according to their knee symptoms. Thus, the patients were informed about joint and knee osteoarthritis, the signs and symptoms of the disease and the type of daily care that they should have. They were also instructed to use ice packs if they had any swelling of the knee and hot packs if they had any soreness without swelling. The verbal and written explanation used simple and comprehensible language.

### Outcome assessment

The pre and post-intervention evaluations were done by a physiotherapist who was blind to the particular treatment used. The outcomes used in our study were based on previous studies on patients with knee osteoarthritis. To define the outcomes used, it was assumed that the interventions of the study would influence them similarly. That similarity was based on a previous knee osteoarthritis rehabilitation study.^22^ The Committee for Proprietary Medicinal Products (CPMP) has recommended that in defining the outcomes, only those that the intervention is found to influence in a similar manner should be included. Inclusion of an outcome that does not have the capability to detect the effects of a treatment can lead to increased variability and, thus, decreased sensitivity towards demonstrating the real difference between the treatments.^22^

### Primary outcomes

The primary outcomes were analyzed through the TUG test and the NRS for pain intensity. The TUG test is a simple and inexpensive method that was developed to assess functional mobility during patients’ daily activities. It comprises the following sequence of movements: standing up from a seated position, walking three meters, turning around, walking back, and sitting down again. The time that the patient takes to perform this sequence of movements is recorded, so that it can be compared before and after the treatment.^8^ In our study, the patients practice before the test was recorded. The best result after three attempts was used as the final result. In the NRS, the participants were asked to score their pain (0 to 10) when walking on a flat surface.^23^ All the questionnaires used were versions that had been translated into and validated for Portuguese.^24^^,^^25^^,^^26^

### Secondary outcomes

The secondary outcomes used were the Lequesne index and the ADL scale.^6^^,^^7^ The Lequesne index is a ten-question survey given to patients suffering from knee osteoarthritis. It is composed of five questions relating to pain or discomfort, one question dealing with the maximum distance walked and four questions about activities of daily living. The total questionnaire is scored on a scale from 0 to 24. Higher scores indicate that there is greater functional impairment. A study by Faucher et al.^6^ found that the Lequesne index was a reliable questionnaire.

Irrgang et al.^7^ developed and validated the ADL scale. The purpose of this questionnaire is to measure the functional capacity and symptoms of the knee and what impact these have on patients’ daily lives. It is composed of 14 items in which patients are asked about symptoms of instability, stiffness, weakness and knee swelling. This questionnaire includes questions about their ability to walk up and down stairs, squat and kneel. The maximum score is 70, but this value is usually converted into a percentage (0-100%).

### Statistical analysis

To analyze the effects, the changes to the scores were calculated (follow-up score minus baseline score). To calculate the difference between the groups, we used the principle of analysis by intention to treat (ITT). In the ITT analysis, we used a mixed-model repeated-measures analysis of variance (ANOVA) with measurement occasion as a within-group factor and the intervention as a between-group factor. Relationships between observations on different occasions were modeled as an unstructured covariance matrix. No *ad hoc* imputation was performed to evaluate the differences in changes from the baseline between each of the three conditions. The ITT analyses used differed from the previously published analysis of post-test data in which observations that were missing from post-test evaluations were input by assuming that participants who withdrew were unchanged. Chakraborty and Gu showed that mixed-model analysis without any *ad hoc* imputation always provides equal or more power than an analysis using mixed models with missing values imputed ad hoc.^27^ The effect size was computed as the difference between the means, divided by the pooled standard deviation, using Cohen’s d.^28^ These analyses were undertaken using general linear model (GLM) and mixed procedures in the statistical analysis software (SAS) release 9.2 for Windows. P values < 0.05 denote a statistically significant difference in this study.

## RESULTS

### Participants

**Figure 1.**
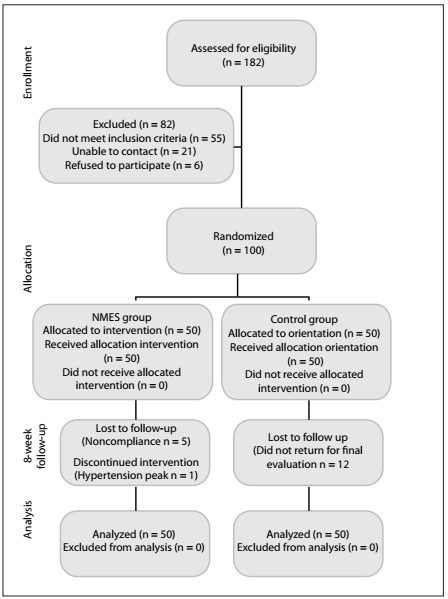
Flowchart of the patients randomized and analyzed per group.

The demographic characteristics of the patients regarding gender, treated leg, age, body mass index (BMI) and baseline values for the NRS, TUG test, Lequesne index and ADL scale are described in Table 1. Eighty-two patients completed the study. The percentages that failed to complete for each group were 12% (n = 44) in the NMES group and 24% (n = 38) in the control group **(**Figure 1**)**.

**Table 1.** Summary of participant demographics and mean baseline values for outcomes

Variable	CG	NMESG	P-value
Gender (%)			
Female	94	92	1.00
Male	6	8	
Treated leg (%)			
Right	34	44	0.55
Left	41.67	33.33	
Both sides	22.92	47.92	
Age (years): mean (SD)	58.78 (9.60)	60.60 (6.72)	0.36
BMI: mean (SD)	30.00 (5.05)	30.08 (3.80)	0.95
KL grade (%)			
2	91.18	95.35	0.82
3	5.88	2.33	
4	2.94	2.33	
Medication usage (8 weeks): mean (SD)	7.71 (13.69)	5.61 (9.35)	1.00
NRS (0-10): mean (SD)	6.92 (2.60)	7.06 (1.95)	0.97
TUG test: mean (SD)	10.08 (2.96)	8.27 (1.76)	0.00
Lequesne index: mean (SD)	13.39 (3.26)	12.33 (3.84)	0.14
ADL scale: mean (SD)	50.61 (17.15)	52.75 (16.11)	0.52

SD = standard deviation, CG = control group, NMESG = neuromuscular electrical stimulation group, BMI = body mass index, KL = Kellgreen Lawrence; NRS = numerical rating scale; TUG = timed up and go test; ADL scale = activities of daily living scale.

### Results for the primary and secondary outcomes

After eight weeks, the NMES group showed a statistically significant improvement in NRS (P < 0.0001), TUG test (P < 0.0001), Lequesne index (P < 0.0001) and ADL scale (P < 0.0001). In the control group, the changes in NRS, TUG test, Lequesne index and ADL scale were not statistically significant (P > 0.05) **(**Table 2**)**.

**Table 2.** Results before and after intervention

Outcomes	Groups	In 8^th^ week	Mean difference (95% CI)	P-value within group
NRS (0-10): mean (SD)	CG	5.74 (3.14)	-0.88 (-1.92 to 0.15)	0.09
	NMESG	4.30 (3.01)	-2.70 (-3.56 to -1.84)	< 0.0001
TUG test: mean (SD)	CG	9.22 (3.31)	-0.57 (-1.20 to 0.06)	0.07
	NMESG	6.77 (1.08)	-1.36 (-1.84 to -0.87)	< 0.0001
Lequesne index: mean (SD)	CG	11.76 (4.04)	-1.26 (-2.49 to -0.03)	0.04
	NMESG	8.95 (4.69)	-3.36 (-4.5 to -2.14)	< 0.0001
ADL scale: mean (SD)	CG	55.95 (19.35)	2.79 (-4.31 to 9.90)	0.42
	NMESG	67.92 (17.92)	15.76 (10.33 to 21.18)	< 0.0001

CI = confidence interval; CG = control group; NMESG = neuromuscular electrical stimulation group; NRS = numerical rating scale; TUG = timed up and go test; ADL scale = activities of daily living scale.

The comparison between groups in the ITT analysis showed that there were statistically significant differences in favor of the NMES group regarding the NRS (P = 0.01), ADL scale (P = 0.01) and Lequesne index (P = 0.03) **(**Table 3**)**. No statistically significant difference was found in relation to the TUG test, although there was a statistical trend with P = 0.05.

**Table 3.** Comparison between groups according to the intention-to-treat analysis

Outcome	Difference between means (95% CI)	Effect size (95% CI)	P-value
NRS (0-10)	1.67 (0.31 to 3.02)	0.63 (0.17 to 1.08)	0.01
TUG test	0.73 (-0.00 to 1.47)	0.46 (0.01 to 0.91)	0.05
Lequesne index	1.98 (0.15 to 3.79)	0.55 (0.09 to 1.00)	0.03
ADL scale	-11.23 (-19.88 to -2.57)	-0.58 (-1.04 to -0.12)	0.01

CI = confidence interval; CG = control group; NMESG = neuromuscular electrical stimulation group; NRS = numerical rating scale; TUG = timed up and go test; ADL scale = activities of daily living scale.

### Adverse side effects

An adverse side effect consisting of a hypertensive crisis was experienced by one patient in the NMES group. This may have been due to the use of NMES or to the exercise itself. The contraindications for using NMES were respected, i.e. avoiding the abdomen of pregnant women, areas with tumors, pacemakers, tissue bleeding and areas of active epiphysis.^29^

## DISCUSSION

This study aimed to evaluate the effect of NMES relating to reduction of pain intensity and improvement of mobility, through assessment of pain intensity, functional tests and functional questionnaires. In accordance with our hypothesis, this clinical trial showed that NMES was effective with regard to improvement of pain, function and activities of daily living among patients with knee osteoarthritis.

The study of Talbot et al.^14^ compared home-based NMES for the quadriceps muscle with an education group. Post-intervention, the pain intensity decreased and the functional tests showed improvements in both groups, but no significant difference was observed between the groups. In our study, we did not find any improvement in any outcome in the control group, which only received educational guidance. The difference in results between our study and the study by Talbot et al.^14^ was possibly because, in their study, NMES was applied at home without supervision by a professional. Thus, factors such as adherence to treatment and differences in electrode positioning and intensity of electric current may have altered the outcome.

In the study by Palmieri-Smith et al.,^13^ a group that received NMES was compared with a group that received no treatment or guidance. Similarly to our results, there was a statistically significant difference in the degree of pain reduction, an improvement in function (only in the 16^th^ week) and no statistically significant difference between the groups regarding improvement in performance tests in the 5^th^ or 16^th^ week.

As mentioned earlier, the TUG test assessment in the present study did not show any statistically significant difference in comparison with the controls in the ITT analysis. Given that the TUG test produced P = 0.05, it is possible that a larger sample size would have shown P < 0.05. In addition, because the patients did not present any significant functional impairment, i.e. there was no significant limitation in relation to walking or getting up from and sitting down on a chair, it is possible that the TUG test was unable to effectively detect changes in mobility among patients that functionality questionnaires detect. Performance tests do not necessarily reflect aspects of individual mobility, because they are isolated activities. Therefore, the time or distance results recorded using the tests performed need to be assessed in association with functionality questionnaires and should not be analyzed in isolation.^30^ According to the study by Wright et al.,^17^ among the four tests that assessed performance (40-m self-paced walk test, 30-s chair-stand and 20-cm step test), only the TUG test did not detect any statistically significant improvement in the group of patients who were examined using the global change rating score (GCRS), among those who reported improvement after the treatment.

A systematic review published in the Cochrane Library assessed the effectiveness of NMES applied before and after total knee arthroplasty. It was deduced, based on the results from two studies, that no conclusion could be reached regarding the effectiveness of NMES among this group of patients. It is also worth noting that these two studies presented a high risk of methodological bias.^31^ Another comprehensive study aimed to investigate the effectiveness of NMES after surgery to reconstruct the anterior cruciate ligament.^32^ Based on eight studies that were included in the analysis, it was concluded that NMES combined with exercises can be effective in improving quadriceps strength within the first four weeks post-operation. It is considered that NMES may have beneficial effects in terms of improving patients’ functional capabilities, but more studies are needed in order to provide a more accurate conclusion. With regard to functional performance tests, there is insufficient evidence to indicate whether NMES has positive or negative effects on functional performance among such patients.^33^

The statistical analysis used in this study was performed using the intention-to-treat principle. ITT analysis takes into account all randomized patients. When ITT analysis is performed, the protocol violations that occur after randomization and might have an impact on the data and conclusions are minimized. Thus, ITT analysis should be performed because it avoids overestimation of the effect.^17^ According to CONSORT,^17^ ITT analysis is widely recommended as the preferred analysis strategy.

One possible limitation of the present study was the lack of registration of NMES intensity. However, the patients were often asked whether the intensity could be increased. Therefore, in accordance with previous studies,^11^^,^^15^ we used the maximum intensity tolerated by the patients.

## CONCLUSION

NMES, within a rehabilitation protocol for patients with knee osteoarthritis, is effective with regard to improving pain, function and activities of daily living among these patients.
